# A Unified Deep Learning Framework for Single-Cell ATAC-Seq Analysis Based on ProdDep Transformer Encoder

**DOI:** 10.3390/ijms24054784

**Published:** 2023-03-01

**Authors:** Zixuan Wang, Yongqing Zhang, Yun Yu, Junming Zhang, Yuhang Liu, Quan Zou

**Affiliations:** 1 School of Computer Science, Chengdu University of Information Technology, Chengdu 610225, China; 2 Institute of Fundamental and Frontier Sciences, University of Electronic Science and Technology of China, Chengdu 610054, China

**Keywords:** single-cell ATAC-seq analysis, ProdDep Transformer Encoder, chromatin accessibility prediction, cell type annotation, scATAC-seq data denoising, TF activity inference

## Abstract

Recent advances in single-cell sequencing assays for the transposase-accessibility chromatin (scATAC-seq) technique have provided cell-specific chromatin accessibility landscapes of cis-regulatory elements, providing deeper insights into cellular states and dynamics. However, few research efforts have been dedicated to modeling the relationship between regulatory grammars and single-cell chromatin accessibility and incorporating different analysis scenarios of scATAC-seq data into the general framework. To this end, we propose a unified deep learning framework based on the ProdDep Transformer Encoder, dubbed PROTRAIT, for scATAC-seq data analysis. Specifically motivated by the deep language model, PROTRAIT leverages the ProdDep Transformer Encoder to capture the syntax of transcription factor (TF)-DNA binding motifs from scATAC-seq peaks for predicting single-cell chromatin accessibility and learning single-cell embedding. Based on cell embedding, PROTRAIT annotates cell types using the Louvain algorithm. Furthermore, according to the identified likely noises of raw scATAC-seq data, PROTRAIT denoises these values based on predated chromatin accessibility. In addition, PROTRAIT employs differential accessibility analysis to infer TF activity at single-cell and single-nucleotide resolution. Extensive experiments based on the Buenrostro2018 dataset validate the effeteness of PROTRAIT for chromatin accessibility prediction, cell type annotation, and scATAC-seq data denoising, therein outperforming current approaches in terms of different evaluation metrics. Besides, we confirm the consistency between the inferred TF activity and the literature review. We also demonstrate the scalability of PROTRAIT to analyze datasets containing over one million cells.

## 1. Introduction

The single-cell sequencing assay for transposase-accessible chromatin (scATAC-seq) reveals chromatin accessibility landscapes of cis-regulatory elements at a single-cell resolution [1]. The scATAC-seq assay has been successfully applied to annotate cell types [2], infer the activity of transcription factors (TFs) [3], and reconstruct cell differentiation trajectories [4]. However, the inherent high dimensionality of accessible cis-regulatory elements [5] and the sparsity of sequence reads per cell [6,7] present a unique challenge for analyzing scATAC-seq data. Therefore, developing computational approaches to represent the scATAC-seq data has become essential in bioinformatics.

In recent years, many algorithms have been designed to cope with high-dimensional and sparse single-cell sequencing data, especially single-cell RNA-seq (scRNA-seq) data. To reduce the dimension of scRNA-seq data, many techniques such as principal component analysis (PCA) [8], t-distributed stochastic neighbor embedding (t-SNE) [9], and uniform manifold approximation and projection (UMAP) [10] are employed to map raw data into a lower dimensional space. To overcome the sparsity of scRNA-seq data caused by the missing expression values, many imputation techniques are proposed to recover dropout values. For example, SAVER [11], an expression recovery model for scRNA-seq, borrows information from similar cells and genes to fill in missing transcripts. MAGIC [12] recovers undetected gene expressions in scRNA-seq data by the Markov affinity-based graph imputation. However, direct applications of the aforementioned scRAN-seq analysis approaches to scATAC-seq data may not be suitable due to the higher dimension and severer sparsity of the scATAC-seq data.

Recently, several approaches have been specially developed for scATAC-seq data analysis, which can be broadly categorized into sequence-free and sequence-dependent methods. From the peak-by-cell matrix, sequence-free approaches represent annotated peaks as genomic coordinates and characterize individual cells by detecting the biologically significant covariance. For example, cisTopic [13] and SCALE [14] use Latent Dirichlet Allocation or a variational autoencoder to capture latent features that characterize the distributions of scATAC-seq data generated on different platforms with different protocols. However, sequence-free approaches ignore sequence information and rely on post hoc algorithms to relate to cis-regulatory factors. Sequence-dependent approaches further improve the performance of scATAC-seq analysis by considering sequence features, such as the k-mer content or the occupancy of the TF-DNA binding motif. For instance, BROCKMAN [15] represents each peak as a set of DNA words (k-mer) and aggregates the k-mer frequency across peaks to learn cell representations. scBasset [16] is a deep convolution neural network to predict single-cell chromatin accessibility from the DNA sequence, which regards the weights of the final layer as the cell representations. Nevertheless, the sequence-dependent above approaches need to consider more regulatory grammars, such as the position and long-range dependency of motifs, for improving accuracy and interpretability. In addition, it is a challenge to incorporate various analysis scenarios, such as chromatin accessibility prediction, cell type annotation, scATAC-seq data denoising, and TF activity inference, into a general computational framework.

**Figure 1:**
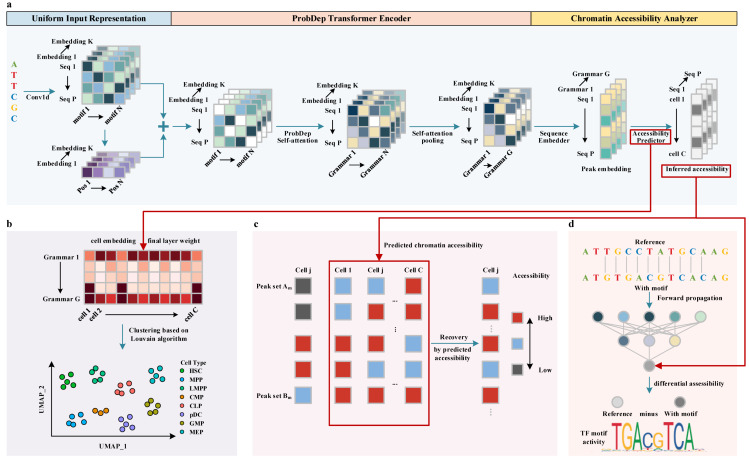
The overview of PROTRAIT. (**a**) Chromatin accessibility modeler. This module comprises a Uniform Input Representation, a ProbDep Transformer Encoder, and a Chromatin Accessibility Analyzer. Specifically, Uniform Input Representation encodes each regulatory motif’s occupancy and position information. ProbDep Transformer Encoder further captures bona fide long-range dependency between different regulatory motifs. Chromatin Accessibility Analyzer integrates learned regulatory grammars to predict the chromatin accessibility of each cell. The final layer weight of the Chromatin Accessibility Analyzer can be regarded as cell embedding. (**b**) Cell type annotator. After generating cell embedding, this module utilizes the Louvain algorithm to perform single-cell clustering for cell type annotation. (**c**) scATAC-seq data denoiser. This module utilizes a statistical model to discover which zero counts are affected by dropout events. It only performs recovery on discovered zero counts by predicted chromatic accessibility. (**d**). TF activity analyzer. This module analyzes the activity of the TF in each cell or nucleotide by measuring changes in predicted accessibility.

Here, we present PROTRAIT, a unified deep learning framework based on ProdDep Transformer Encoder for analyzing scATAC-seq data. Our framework comprises four parts: (i) Chromatin accessibility modeler. It utilizes the ProdDep Transformer Encoder to capture the occupancy, position, and long-range dependency of motifs from DNA sequence for predicting single-cell chromatin accessibility and learning a latent embedding of each cell; (ii) Cell type annotator. Based on the learned cell embedding, it utilizes the Louvain algorithm to perform clustering for annotating the types of each cell; (iii) scATAC-seq data denoiser. It uses a statistical model to automatically identify likely noises and perform denoising on these values by predicted accessibility; and (iv) TF activity analyzer. Feeding synthetic DNA sequences, it analyzes the activity of the TF in each cell or nucleotide by measuring changes in predicted accessibility. The overview of PROTRAIT is shown in Figure 1. Experimental results on scATAC-seq datasets from the Buenrostro2018 study demonstrate that PROTRAT achieves state-of-the-art performance across various analysis scenarios, including chromatin accessibility prediction, cell type annotation, scATAC-seq data denoising, and TF activity inference. Besides, we demonstrate the scalability of PROTRAIT to analyze datasets containing over one million cells.

## 2. Results and Discussion

### 2.1. PROTRAIT Predicts Single-Cell Chromatin Accessibility on Held-Out DNA Sequences

**Figure 2:**
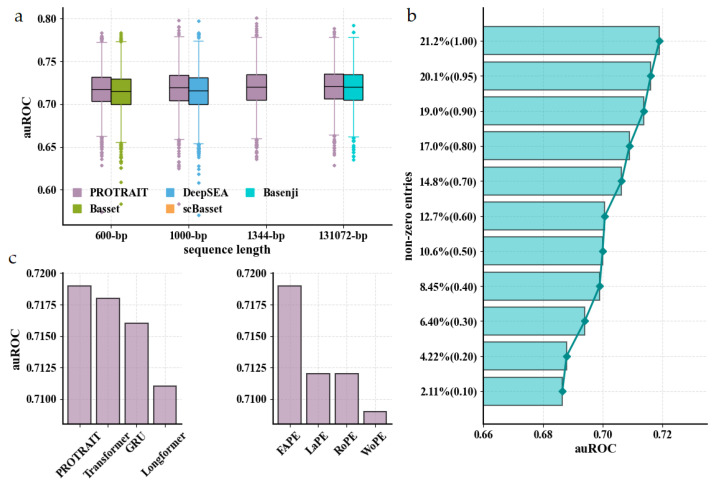
Evaluation of chromatin accessibility prediction approaches for scATAC-seq data. (**a**) auROC comparison of PROTRAIT and different chromatin accessibility prediction approaches, including Basset, DeepSEA, scBasset, and Basenji. (**b**) auROC comparison of PROTRAIT on down-sampled datasets (non-zero entries of labels are 95%, 90%, 80%, 70%, 60%, 50%, 40%, 30%, 20% and 10% of original). (**c**) auROC comparison of the ProbDep Transformer Encoder and its variants (**left**) and the fixed absolute position embedding and its variants (**right**).

We first investigate whether PROTRAIT accurately predicts chromatin accessibility across cells for held-out DNA sequences, guaranteeing that PROTRAIT has learned a meaningful relationship between DNA sequence and chromatin accessibility. For held-out DNA sequences in Buenrostro 2018 dataset, we compute the area under the receiver operating characteristic curve (auROC) of PROTRAIT, Basset [17], DeepSEA [18], scBasset [16], and Basenji [19]. Basset, DeepSEA, scBasset, and Basenji are well-verified chromatin accessibility analyzers based on convolutional neural networks and receive DNA sequences of 600 bp, 1000 bp, 1344 bp, and 131072 bp as input, respectively. To make a fair comparison, we utilize DNA sequences of 600 bp, 1000 bp, 1344 bp, and 131072 bp, respectively, as input of PROTRAIT. PROTRAIT substantially outperforms all competitors across different input lengths (Figure 2a) because PROTRAIT can simultaneously learn occupancy, position, and long-range dependency of TF-DNA binding motifs. However, Basset, DeepSEA, and Basenji performances are slightly below the 0.75–0.95 range achieved for bulk chromatin accessibility samples in original publications because of dropouts in the scATAC-seq technique, causing the sparse and noisy labels. To further assess the influence of the sparse and noisy labels, we randomly down-sample 95%, 90%, 80%, 70%, 60%, 50%, 40%, 30%, 20% and 10% non-zero entries from original labels as new labels and run the PROTRAIT. The auROC of PROTRAIT decreases with the decreasing number of non-zero entries. Still, the performance is acceptable even when the label contains only 10% non-zero entries (Figure 2b). Overall, these results confirm that the PROTRAIT architecture advances prediction accuracy and robustness for single-cell chromatin accessibility.

To pinpoint the benefit of the ProbDep Transformer Encoder, we replace it with the gated recurrent unit (GRU) [20], the canonical Transformer Encoder [21]. The Longformer Encoder [22], respectively. The GRU and canonical Transformer Encoder capture long-range dependency between any two motifs in a whole DNA sequence, whereas Longformer Encoder only focuses on such dependence surrounding each motif. ProbDep Transformer Encoder consistently outperforms the GRU, the Transformer Encoder, and the Longformer Encoder (Figure 2c left), demonstrating the utility of learning bona fide long-range dependencies with biological significance, for example, the dependencies between the motifs of TFs and their cofactors. Then, we assess the effect of position information of regulatory motifs on the ProbDep Transformer Encoder. The fixed absolute position embedding (FAPE) [21] yields better performance than the learnable position embedding (LaPE) [23], and the rotation position embedding (RoPE) [24] (Figure 2c right), which is because makes the model easy to converge. The capability to consider position information of regulatory motifs is indeed crucial because we observe a significant performance drop when using the model without position embedding (WoPE) (Figure 2c right). These results confirm that ProbDep Transformer Encoder is suited for chromatin accessibility prediction.

### 2.2. PROTRAIT Annotates Cell Types by Clustering on Cell Embedding

**Figure 3:**
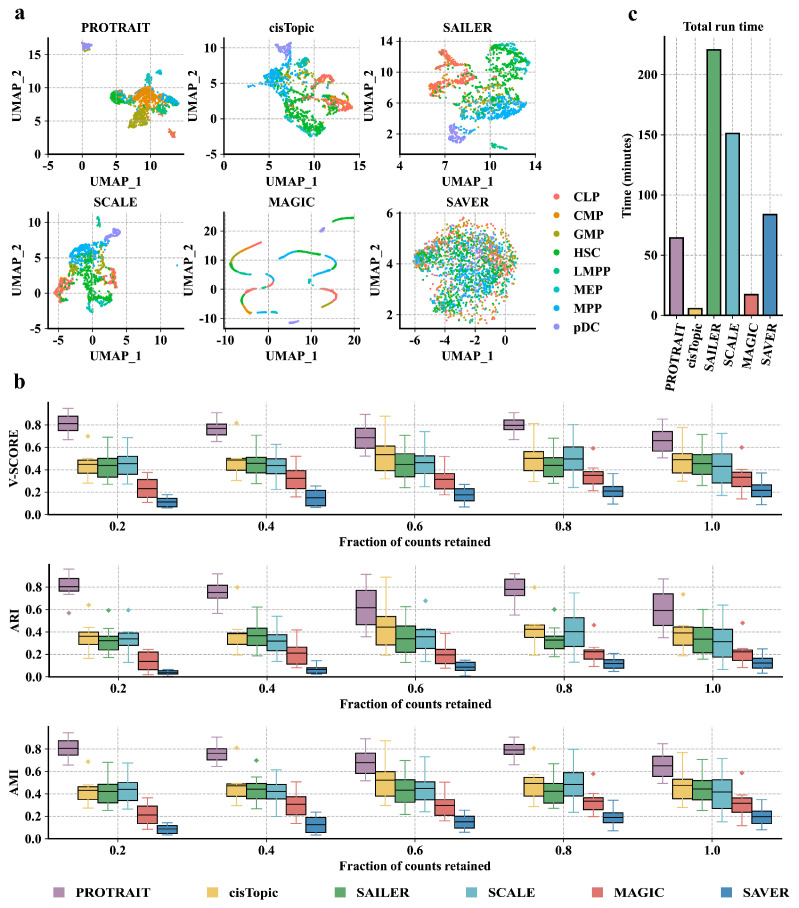
Performance of diverse cell representation approaches compared by cell type annotation. (**a**) UMAP representation of the cell embeddings derived from PROTRAIT, cisTopic, SAILER, SCALE, MAGIC, and SAVER. All approaches are trained on the full dataset. (**b**) The average *v*-score, ARI, and AMI for each cell type are measured after clustering based on the cell embeddings. All approaches are trained on the datasets where the total number of peaks per cell remains at 20%, 40%, 60%, 80%, and 100% of the total peaks, respectively. (**c**) Runtimes for each of the cell-embedding approaches profiled. All approaches are trained on the full dataset.

We begin by examining the ability of PROTRAIT to generate the cell embedding that characterizes scATAC-seq data distribution. Specifically, we (i) run PROTRAIT, cisTopic, SAILER [5], SCALE, MAGIC, and SAVER based on the scATAC-seq data of eight homogenous cell types in the HSC differentiation lineage, including common lymphoid progenitor (CLP), common myeloid progenitor (CMP), granulocyte-macrophage progenitors (GMP), hematopoietic stem cell (HSC), lymphoid-primed multipotent progenitor (LMPP), megakaryocyte-erythrocyte progenitor (MEP), multipotent progenitor (MPP), and plasmacytoid dendritic cell (pDC); and subsequently (ii) visualize the generated cell embeddings from approaches above with the UMAP. The cell embeddings of PROTRAIT, cisTopic, SAILER, and SCALE are better separated between cell types. In contrast, the embeddings of MAGIC and SAVER overlapped between some cell types (Figure 3a) because approaches specially designed for scRNA-seq data hardly fit higher dimensional and sparser scATAC-seq data. Furthermore, PROTRAIT can also reveal the development trajectory of different cell types in UMAP visualization (Figure 3a). For example, LMPP and CMP cells are close to MPP cells in the embedding, which is consistent with the differentiation lineage diagram of the HSC [25]. These results confirm that PROTRAIT can generate the cell embedding that characterizes scATAC-seq data distribution.

We further assess the correctness of PROTRAIT in annotating cell types. Specifically, we (i) randomly down-sample 100%, 80%, 60%, 40% and 20% peaks from the original dataset and run PROTRAIT, cisTopic, SAILER, SCALE, MAGIC, and SAVER based on the down-sampled data; and subsequently (ii) apply Louvain clustering on the generated cell embeddings from these approaches and compute the adjusted rand index (ARI), adjusted mutual information (AMI) and *v*-measured score (*v*-score) by comparing clustering results with ground-truth cell-type labels. PROTRAIT displays the best ARI, AMI, and *v*-score on all five different sampling frequencies (Figure 3b), which demonstrates the effectiveness of PROTRAIT in annotating cell types. Besides, ARI, AMI, and *v*-score increase under some sampling frequencies because some peak sequences may be non-functional in cis-regulatory and cell development. These results confirm that PROTRAIT can accurately annotate cell types by clustering on its generated cell embedding.

We finally explore the runtime of different cell representation approaches. PROTRAIT, cisTopic, SAILER, SCALE, MAGIC, and SAVER consumes 64.3, 12.1, 220.6, 151.2, 17.2, and 83.8 minutes to reach convergence, respectively (Figure 3c). The runtimes for SCALE, SAILER, and PROTRAIT can be further improved by using a graphics processing unit with sufficient memory due to the nature of deep learning. Besides, designing lightweight architectures to replace the original ones for scATAC-seq data analysis is another promising direction for improving the runtime.

### 2.3. PROTRAIT Denoises Single-Cell Chromatin Accessibility Profiles

**Figure 4:**
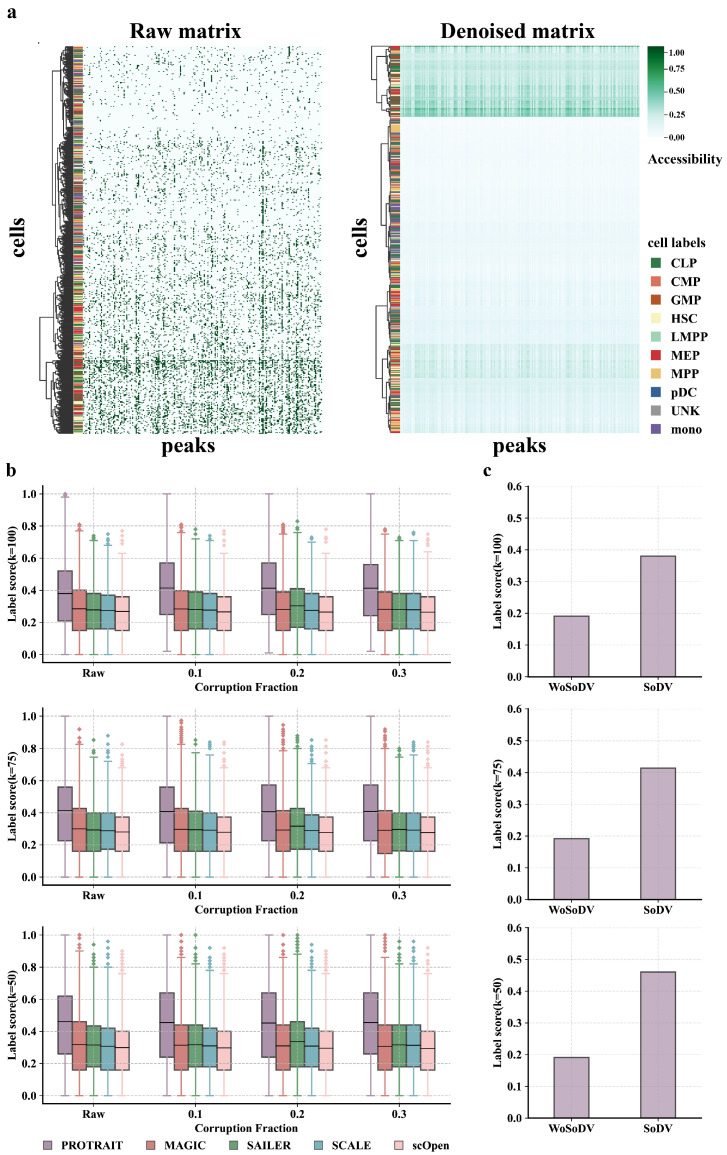
Performance evaluation of denoising approaches for scATAC-seq data. (**a**) The raw and denoised peak-by-cell matrix of 800 peaks and 200 cells from the Buenrostro2018 dataset, hierarchically clustered by cells. (**b**) The label score (k = 50, 75, 100) comparison of PROTRAIT, MAGIC, SAILER, SCALE, and scOpen. (**c**) The label score (k = 50, 75, 100) comparison of PROTRAIT with search of dropout values (SoDV) and without search of dropout values (WoSoDV).

The binary accessible indicator for any given cell and peak comprises many false zero counts because of the sparsity of scATAC-seq caused by the experimental noise. To evaluate the ability of PROTRAIT to remove noise on scATAC-seq data, we randomly sample 200 peaks and 500 cells from the Buenrostro 2018 dataset and directly visualize the raw peak-by-cell matrix and the denoised matrix. The cells are clustered by sequencing depth in the raw cell-by-peak matrix (Figure 4a left), showing no biologically significant patterns. After denoising, cells of the same cell type share similar accessibility profiles (Figure 4a right). These results confirm that PROTRAIT can estimate the real scATAC-seq data distribution.

Then, we further corrupt the scATAC-seq data by randomly dropping out peaks at different rates and compare PROTRAIT with MAGIC, SCALE, scOpen [6] and SAILER. We measure the denoising performance by label score, which quantifies what percentage of each cell’s neighbors share the same label in a given neighborhood. In practice, we perform PCA (50 components) on denoising peak-by-cell matrix and use label score (k = 50, 75, 100) to measure whether cells of the same label are embedded closer. Across all corruption rates, PROTRAIT yields better performance than all four state-of-the-art denoising approaches (Figure 4b), which quantitatively indicates the effeteness of PROTRAIT in denoising single-cell chromatin accessibility profiles.

Finally, we verify the effectiveness of identifying likely noises before denoising. Specifically, we perform denoising based on PROTRAIT with and without the search of dropout values and compute the label score. Across most cells, searching for dropout values can significantly improve the performance of single-cell chromatin accessibility data denoising (Figure 4c). This result suggests that PROTRAIT can reduce technical variation from scATAC-seq and avoid introducing excess biases during its denoising process.

### 2.4. PROTRAIT Infers TF Activity at Single-Cell and Single-Nucleotide Resolution

**Figure 5:**
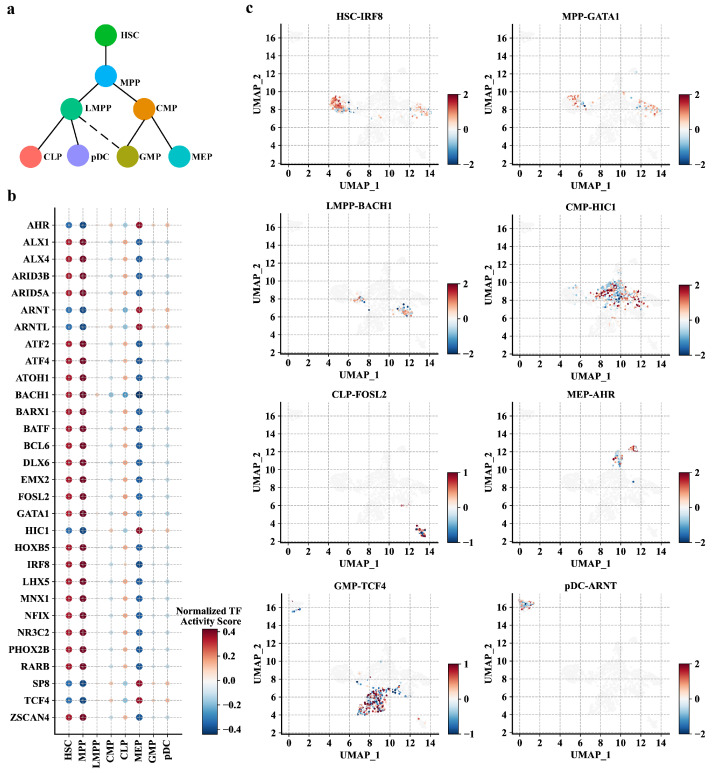
Performance of PROTRAIT in inferring single-cell TF activity. (**a**) HSC differentiation lineage diagram. (**b**) The normalized average activity scores of 30 TFs in eight cell types. (**c**) UMAP represents the single-cell activity scores of the most active TFs in eight cell types.

TF-DNA binding is the primary driver of chromatin accessibility [26,27,28]. As PROTRAIT learns to predict chromatin accessibility from DNA sequences, we expect the framework to capture sequence information predictive of TF-DNA binding. To query the TF activity at single-cell resolution, we (i) perform motif insertion for 30 available human JASPAR motifs [29]; (ii) feed synthetic DNA sequences with and without a particular TF-DNA binding motif of interest to an implemented PROTRAIT framework; and (iii) measure the activity of the motif in each cell based on changes in predicted accessibility. The average TF activity score demonstrates valuable meaning to delineate the differentiation process of HSC (Figure 5a,b). For example, IRF8, a known TF of modulating lineage commitment decisions by HSCs, shows the highest activity in HSCs [30]. GATA1, a key regulator of organizing the hematopoietic lineage fate decision to form the earliest hematopoietic branchpoint, is predicted to be most active in MPPs [31]. BACH1, a master regulator of controlling erythroid-myeloid and lymphoid–myeloid differentiation, has the highest anticipated activity in LMPPs [32]. HIC1, a known TF in myeloid differentiation and survival, shows the most increased activity in CMPs [33]. AHR, a key regulator of regulating the production of bipotential hematopoietic and megakaryocyte-erythroid progenitor, is predicted to be most active in MEPs [34]. TCF4, a part of the leukemia initiation signature in GMPs, has the highest anticipated activity in GMPs [35]. ARNT, a known TF with a specific inhibitory role in pDC biology, shows the most increased activity in pDCs [36]. The relationship between FOSL2 and CLPs now awaits experimental validation. Besides, for a specific cell type, the most active TF shows considerable variation in their activity scores across different single cells (Figure 5c). These results confirm that PROTRAIT can quantify TF activity at single-cell resolution.

**Figure 6:**
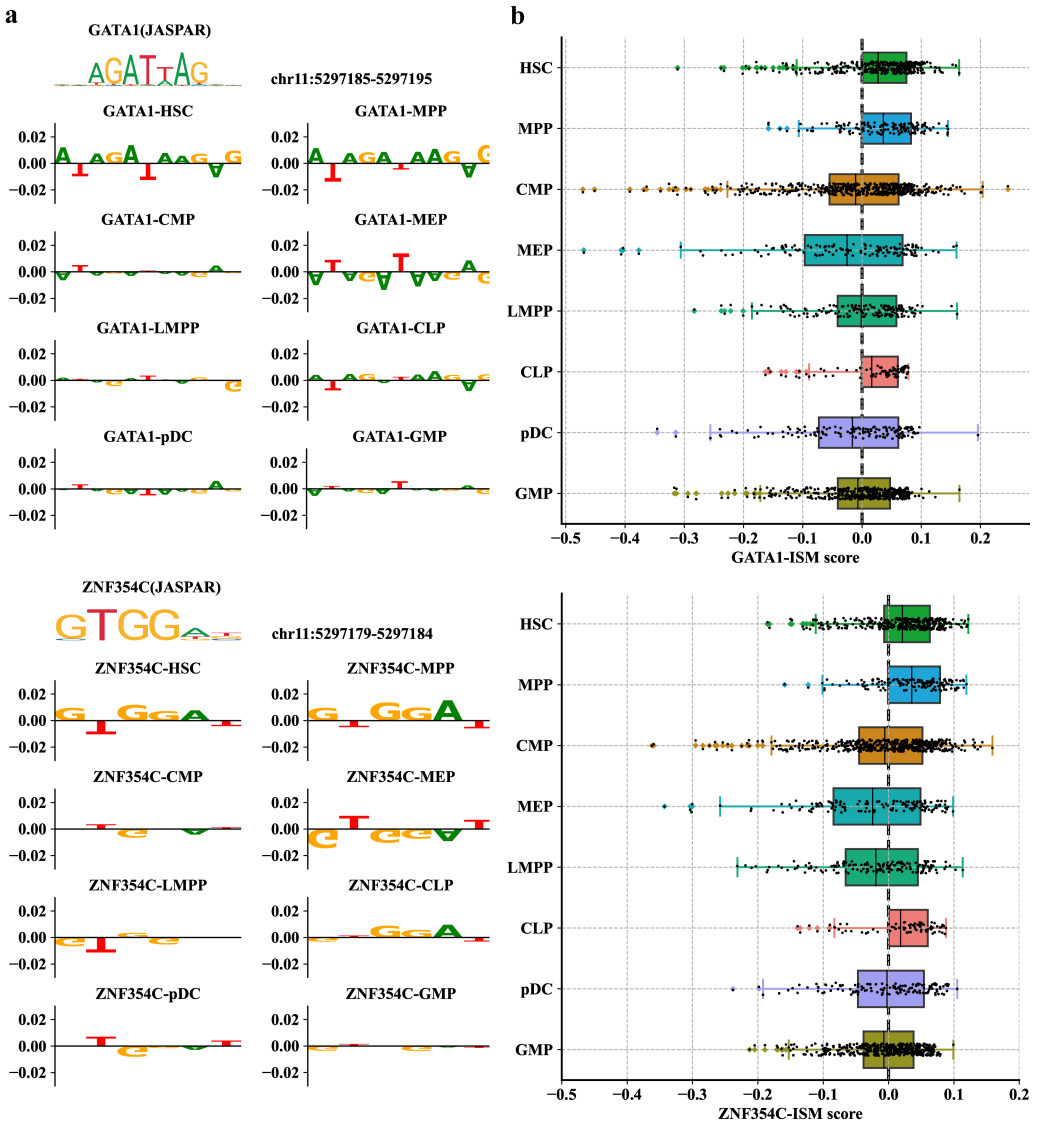
Performance of PROTRAIT in inferring single-cell and single-nucleotide TF activity. (**a**) ISM scores for sequences that match GATA1 and ZNF354C motifs in eight cell types. (**b**) Distributions of single-cell PWM-ISM scores for GATA1 and ZNF354C in eight cell types. The PWM-ISM score is the dot product of the PWM and ISM measurements of motif matches.

To further explore whether PROTRAIT can infer TF activity at both single-cell and single-nucleotide resolution, we (i) performed in silico saturation mutagenesis (ISM) for a known enhancer for the β-globin gene that regulates erythroid-specific β-globin expression; and (ii) mapped the most influential nucleotides into the position weight matrices (PWMs) by the TF-MoDSW algorithm [37]. The most influential nucleotides corresponded to GATA1 and ZNF354C motifs (Figure 6a), which are known to co-bind to β-globin enhancers [38]. Examining the single-cell ISM scores, we also observed that the GATA1 and ZNF354C motifs contribute more to accessibility in HSC, MPP, and CLP cell types. In contrast, the nucleotides of these two motifs have low scores in CMP, MEP, LMPP, pDC, and GMP (Figure 6b). This result demonstrates that TF GATA1 and ZNF354C may be co-active in HSC differentiation. In summary, PROTRAIT can learn regulatory grammar at both single-cell and single-nucleotide resolution and can be used to discover enhancers in individual cells.

### 2.5. PROTRAIT Is Scalable to Large Datasets

**Figure 7:**
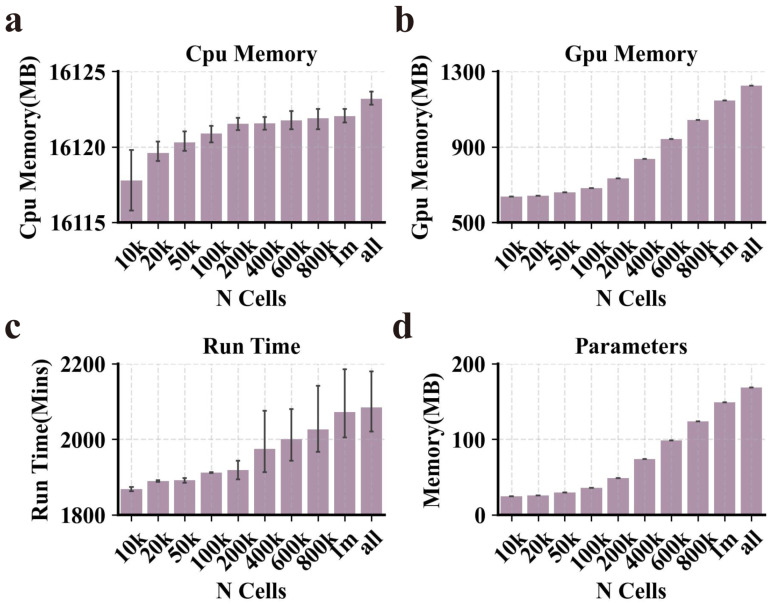
Performance of PROTRAIT in large-scale scATAC-seq analysis. (**a**–**d**) The runtime, peak CPU memory usage, peak GPU memory usage, and parameter of PROTRAIT on sciATAC human atlas. To remove fluctuations from random sampling, we employ a cross-validation strategy and repeat the down-sampling 10 times to obtain averages.

To explore whether PROTRAIT can work for large datasets, we applied our approach to the sci-ATAC human atlas, one of the extensive available scATAC-seq datasets and comprises 1,114,621 cells and 118,043 peaks [39]. Specifically, we ran PROTRAIT on the down-sampled sci-ATAC-seq dataset with 10,000, 20,000, 50,000, 100,000, 200,000, 400,000, 600,000, 800,000, one million, and all cells and measure the runtime, peak CPU memory usage, peak GPU memory usage and parameter (Figure 7). PROTRAIT requires 2084.43 mins on the whole sci-ATAC-seq dataset using an Nvidia GeForce RTX 3080 GPU, with a peak CPU memory usage of 16,123.21 MB, peak GPU memory usage of 1252.73 MB, and parameter of 168.58 MB. More importantly, the runtime, peak CPU memory usage, and peak GPU memory usage only increase slightly with the number of cells. When we increase the number of cells from 10,000 to 1,114,621 (100×), the runtime goes from 1868.14 mins to 2084.43 mins (1.12×), CPU memory goes from 16,119.78 MB to 16,123.21 MB (1.01×), and GPU memory goes from 651.61 MB to 1252.73 MB (1.92×). The parameter goes from 24.49 MB to 168.58 MB (6.88×). These results suggest that PROTRAIT is suitable for analyzing a sizeable scATAC-seq compendium.

## 3. Materials and Methods

### 3.1. Datasets

We downloaded the processed h5ad file for Buenrostro 2018 generated by Yuan et al. at http://storage.googleapis.com/scbasset_tutorial_data/buen_ad_sc.h5ad (accessed date 23 December 2022) [16]. The Buenrostro 2018 dataset comprises 10 fluorescence-activated cell-storing (FACS) cell populations from CD34+ human bone marrow, namely, CLP, CMP, GMP, HSC, LMPP, MEP, MPP, pDC, monocytes (mono), and an uncharacterized CD34+, CD38-, CD45RA+, CD123- cell population. A total of 2034 cells and 31,783 peaks from six human donors were used for analysis.

### 3.2. Chromatin Accessibility Modeler Based on ProbDep Transformer Encoder

The chromatin accessibility modeler is a deep language model for predicting single-cell chromatin accessibility from the DNA sequence, which comprises a Uniform Input Representation, a ProbDep Transformer Encoder, and a Chromatin Accessibility Analyzer. Specifically, Uniform Input Representation encodes each regulatory motif’s occupancy and position information. ProbDep Transformer Encoder further captures bona fide long-range dependency between different regulatory motifs. The Chromatin Accessibility Analyzer first integrates learned regulatory grammars to generate explicit embedding of thev DNA sequence. Then it utilizes a linear layer to transform the sequence embedding for predicting the chromatin accessibility of each cell. The parameters of the linear layer can be regarded as the cell embedding, which indicates how much cell-specific chromatin accessibility depends on each regulatory grammar. In summary, the chromatin accessibility modeler can serve as a single-cell chromatin accessibility predictor and a representation learning machine for each cell.

#### 3.2.1. Uniform Input Representation

Uniform Input Representation comprises Motif Embedding and a Position Embedding. Motif Embedding and Position Embedding represent the occupancy and position of each regulatory motif, respectively.

**Motif Embedding.** Given an *L*-bp DNA sequence, we mapped it into a latent space by using one-hot embedding. Suppose *L* is less than a manually set threshold. In that case, we transformed the one-hot embedding into the motif embedding by a convolutional layer to represent the occupancy of the regulatory motif. Suppose *L* is more significant than a manually set threshold. In that case, we used sequential alternating convolution and pooling layers to generate the motif embedding to reduce the dimension of the embedding.

**Position Embedding.** To make use of the position information of each regulatory motif, we used fixed absolute position embedding:

(1) $${\mathrm{PE}}_{\left(pos,2i\right)}=\sin\left(\frac{pos}{L^{\frac{2i}{d}}}\right)$$

(2) $${\mathrm{PE}}_{\left(pos,2i+1\right)}=\cos\left(\frac{pos}{L^{\frac{2i}{d}}}\right)$$

where pos indicates the position of the regulatory motif and *i* denotes the dimension of the position embedding.

After obtaining the motif embedding and the position embedding, we directly summed them to generate the uniform input representation $X^{\left(0\right)}$:

(3) $$X^{\left(0\right)}=\alpha u+{\mathrm{PE}}_{\left(,\right)}$$

where u indicates the motif embedding and α denotes the factor balancing the magnitude between the motif embedding and the position embedding.

#### 3.2.2. ProbDep Transformer Encoder

The transformer Encoder can utilize the self-attention mechanism to compute a weighted sum across the representations of all regulatory motifs for learning each motif’s high-order features. The attention weight between any two regulatory motifs depends on their representations and distances, which allows the model to capture possible long-range dependency. However, long-range dependency only occurs between a small fraction of regulatory motif pairs, such as motifs of a TF and its cofactor. This leads to the sparse distribution of the self-attention mechanism, that is, a few query–key pairs contribute to the major attention, and others generate minor attention. Hence, we developed a ProbDep Transformer Encoder to capture the bona fide long-range dependency, that is, the query–key pair contributing to the major attention, for improving robustness and computational efficiency. The ProbDep Transformer Encoder comprises the long-range dependency measurement, the ProbDep self-attention, and the self-attention pooling. To further discuss the ProbDep Transformer Encoder, let $q_{i}$, $k_{i}$, $v_{i}$ stand for the *i*-th row in Q, K, V, respectively, where Q indicates the query, K denotes the key and V refers to the value.

**Long-range dependency measurement.** The attention of the *i*-th query on all the keys is defined as a probability $p(k_{m}|q_{i})$. The output is its composition with $v_{i}$, where a query–key pair $q_{i}k_{m}^{T}$ indicates possible long-range dependency between two regulatory motifs. A long-range dependency measurement aims to discover bona fide dependency by finding dominant query–key pairs which encourage the attention probability distribution of the corresponding query away from the uniform distribution. If $p(k_{m}|q_{i})$ is close to a uniform distribution $q\left(k_{m}|q_{i}\right)=1/L_{k}$, the self-attention mechanism becomes a meaningless sum of V. We used Kullback–Leibler divergence between $p(k_{m}|q_{i})$ and $q(k_{m}|q_{i})$ to determine the dominant queries for finding the dominate query–key pairs. Dropping constants, the long-range dependency measurement of the *i*-th query is defined as:

(4) $$M\left(q_{i},K\right)=\mathrm{In}\sum_{m=1}^{L_{k}}e^{\frac{q_{i}k_{m}^{T}}{\sqrt{d}}}-\frac{1}{L_{K}}\sum_{m=1}^{L_{K}}\frac{q_{i}k_{m}^{T}}{\sqrt{d}}$$

The first item indicates the Log-Sum-Exp of $q_{i}$ on all the keys, and the second item denotes the arithmetic mean on them. If the *i*-th query gains a larger $M(q_{i},K)$, its attention probability $p(k_{m}|q_{i})$ is more diverse and has a high possibility of containing the dominant query–key pairs. To further improve the efficiency, we used an empirical approximation for the computation of the long-range dependency measurement:

(5) $$\bar{M}\left(q_{i},K\right)=\max_{m}\frac{q_{i}k_{m}^{T}}{\sqrt{d}}-\frac{1}{L_{K}}\sum_{m=1}^{L_{K}}\frac{q_{i}k_{m}^{T}}{\sqrt{d}}$$

In practice, we randomly sampled $L_{K}\mathrm{In}L_{Q}$ query–key pairs to compute $\bar{M}\left(q_{i},K\right)$, that is, filling other pairs with zero, and chose Top-*u* from them as $\bar{Q}$. Controlled by a constant sampling factor *c*, we set $u=c\cdot \mathrm{In}L_{Q}$.

**ProbDep self-attention.** Based on the long-range dependency measurement, we have the ProbDep self-attention by allowing each key to only attend to the *u* dominant queries:

(6) $$A\left(Q,K,V\right)=\mathrm{Softmax}\left(\frac{\bar{Q}K^{T}}{\sqrt{d}}\right)V$$

where $\bar{Q}$ indicates a sparse matrix of the same size as Q, and it only contains the Top-*u* queries under the long-range dependency measurement.

**Self-attention pooling.** To reduce the redundant combinations of $v_{i}$ and make a focused self-attention feature map in the next layer, we developed self-attention pooling. The procedure of self-attention pooling from the *k*-th layer into the (*k*+1)-th layer is defined as:

(7) $$X^{\left(k+1\right)}=\mathrm{MaxPool}\left(\mathrm{Conv}1d\left({\left[X^{\left(k\right)}\right]}_{\mathrm{AB}}\right)\right)$$

where ${\left[\cdot \right]}_{\mathrm{AB}}$ defines the operation of the ProbDep Transformer Encoder layer. Like the Transformer Encoder, the ProbDep Transformer Encoder also adds residual connections and a position-wise feed-forward layer.

#### 3.2.3. Chromatin Accessibility Analyzer

The Chromatin Accessibility Analyzer comprises a Sequence Embedder and an Accessibility Predictor. Specifically, the Sequence Embedder generates a low-dimension sequence representation by integrating the regulatory grammars, including occupancy, position, and long-range dependency of the regulatory motif, from ProbDep Transformer Encoder. The Accessibility Predictor aims to infer cell-specific chromatin accessibility by a linear transformation of sequence representation. The linear transformation’s weight matrix and intercept vector can be regarded as the low-dimension representation and the sequencing depth of cells, respectively.

**Sequence Embedder.** The ProbDep Transformer Encoder’s output $X^{\left(k+1\right)}$ can be regarded as a high-order embedding of the DNA sequence, which has redundant regulatory grammar. To generate a low-redundant sequence embedding Z, Sequence Embedder map $X^{\left(k+1\right)}$ into a low-dimension space by a convolutional layer and a feed-forward layer:

(8) $$Z=\mathrm{ELU}(W_{f}\left(\mathrm{ELU}\left(\mathrm{Conv}1d\left(X^{\left(k+1\right)}\right)\right)\right)+b_{f})$$

where $W_{f}$ and $b_{f}$ indicate the weight matrix and the intercept vector of Sequence Embedder, respectively.

**Accessibility Predictor.** Based on the sequence embedding Z, the Accessibility Predictor determines the accessibility probability in each cell by a linear layer:

(9) $$y=\sigma (ZW_{p}+b_{p})$$

where $W_{p}$ and $b_{p}$ indicate the weight matrix and the intercept vector of Accessibility Predictor, respectively.

**Loss function.** Given the model prediction $y=[y_{1},\ldots ,y_{N}]$ and the binary label $\hat{y}=\left[{\hat{y}}_{1},\ldots ,{\hat{y}}_{N}\right]$, we chose the binary cross-entropy function as the loss function. The loss is propagated back from the Predictor’s output across the entire model:

(10) $$L\left(y,\hat{y}\right)=\frac{1}{N}\sum_{n=1}^{N}y_{n}\log{\hat{y}}_{n}+\left(1-y_{n}\right)\log\left(1-{\hat{y}}_{n}\right)$$

where *N* indicates the number of cells.

#### 3.2.4. Training and Implementation

We monitored the validation loss after every training epoch. Training is stopped early when the validation loss is not decreased in five epochs. This stopping criterion leads to training for around 40 epochs. We used the Adam optimizer to update the model parameters. We randomly searched for hyper-parameters, including mini-batch size and learning rate. The best performance was achieved with a mini-batch size of 16 and a learning rate of 0.001. The chromatin accessibility modeler was implemented based on PyTorch 1.6. All the procedures can be highly efficient vector operations and maintain logarithmic total memory usage.

### 3.3. Cell Type Annotator Based on Cell Embedding

To annotate the cell type of each cell, we performed clustering on the cell embedding. Based on the generated cell embedding, we first created a *k*-nearest neighbor (KNN) graph with neighbors of 15 (implemented by the scanpy in Python), where each cell is represented as a node and edges are drawn between cells within nearest neighbors defined by Euclidean distance. Then, we applied the Louvain algorithm (implemented by the scanpy package in Python), a community discovery algorithm, to detect the communities in the created KNN graph. The communities indicate the groups of cells sharing similar embeddings, possibly originating from the same cell type. Finally, we compare the clustering results to the ground-truth cell type labels through ARI, AMI, and *v*-Score (implemented by the scikit-learn package in Python). To display the results directly, we used UMAP (implemented by the UMAP-learn package in Python) to visualize the cell embedding in the two-dimensional space.

### 3.4. scATAC-Seq Data Denoiser Based on Predicted Chromatin Accessibility

To correct the false zero counts caused by dropout events in scATAC-seq data, we developed a denoising approach that comprises a search and a recovery of dropout values. Specifically, the search for dropout values utilizes a statistical model to discover which zero counts are affected by dropout events. The recovery of dropout values performs recovery on discovered zero counts by using predicted chromatic accessibility.

**Search of dropout values.** Given a peak-by-cell matrix, we employ a probabilistic mixture model to search which peaks are affected by the dropouts in which cells. This model comprises a Gamma distribution and a normal distribution. The Gamma distribution accounts for the dropouts, and the normal distribution indicates the bona fide counts of the scATAC-seq peaks. We separately build probabilistic mixture models for different cell clusters because the proportions of the Gamma distribution and the normal distribution for each peak are different in various cell types.

For each peak *i* in cell type *k*, its count can be modeled as a random variable $X_{i}^{\left(k\right)}$ with the dense function:

(11) $$f_{X_{i}^{\left(k\right)}}\left(x\right)=\lambda_{i}^{\left(k\right)}\mathrm{Gamma}\left(x;\alpha_{i}^{\left(k\right)},\beta_{i}^{\left(k\right)}\right)+\left(1-\lambda_{i}^{\left(k\right)}\right)\mathrm{Normal}\left(x;\mu_{i}^{\left(k\right)},\sigma_{i}^{\left(k\right)}\right)$$

where $\lambda_{i}^{\left(k\right)}$ indicates the overall dropout rate of peak *i* in cell type *k*, $\alpha_{i}^{\left(k\right)}$ and $\beta_{i}^{\left(k\right)}$ denote the shape and rate parameters of the Gamma distribution, $\mu_{i}^{\left(k\right)}$ and $\sigma_{i}^{\left(k\right)}$ refer to the mean and the standard deviation of the normal distribution. The parameters in the probabilistic mixture model are estimated by the Expectation–Maximization algorithm. Thus, the dropout probability of peak *i* in cell *m*, which belongs to *k*, can be inferred as:

(12) $$d_{im}=\frac{{\hat{\lambda }}_{i}^{\left(k\right)}\mathrm{Gamma}\left(X_{im};{\hat{\alpha }}_{i}^{\left(k\right)},{\hat{\beta }}_{i}^{\left(k\right)}\right)}{{\hat{\lambda }}_{i}^{\left(k\right)}\mathrm{Gamma}\left(X_{im};{\hat{\alpha }}_{i}^{\left(k\right)},{\hat{\beta }}_{i}^{\left(k\right)}\right)+\left(1-{\hat{\lambda }}_{i}^{\left(k\right)}\right)\mathrm{Normal}\left(X_{im};{\hat{\mu }}_{i}^{\left(k\right)},{\hat{\sigma }}_{i}^{\left(k\right)}\right)}$$

where $d_{im}$ indicates the dropout probability of peak *i* in cell *m*. ${\hat{\lambda }}_{i}^{\left(k\right)}$, ${\hat{\alpha }}_{i}^{\left(k\right)}$, ${\hat{\beta }}_{i}^{\left(k\right)}$, ${\hat{\mu }}_{i}^{\left(k\right)}$ and ${\hat{\sigma }}_{i}^{\left(k\right)}$ denote the inferred values of $\lambda_{i}^{\left(k\right)}$, $\alpha_{i}^{\left(k\right)}$, $\beta_{i}^{\left(k\right)}$, $\mu_{i}^{\left(k\right)}$ and $\sigma_{i}^{\left(k\right)}$, respectively.

**Recovery of dropout peaks.** For each cell *m*, we first chose peak sets $A_{m}=\left\{i:d_{im}\geq T\right\}$ and $B_{m}=\left\{i:d_{im}<T\right\}$ based on the dropout probabilities of peaks in cell *m*, where *T* indicates a threshold on the dropout probabilities. $A_{m}$ is a set that needs recovery, and $B_{m}$ is a set with an accurate peak count and does not require recovery. Then, for each peak *i*, we gained candidate denoised counts in all cells by predicted chromatic accessibility. Finally, let $r_{i,m}$ stand for the candidate denoised count of peak *i* in cell *m*, and we only recovered the count of peaks in set $A_{m}$:

(13) $${\hat{x}}_{i,m}=\left\{\begin{array}{c} x_{i,m},\;\;\mathrm{if}\;\;i\in B_{m}\\ r_{i,m},\;\;\mathrm{if}\;\;i\in A_{m} \end{array}\right.$$

where $x_{i,m}$ and ${\hat{x}}_{i,m}$ indicate the raw and recovered counts of peak *i* in cell *m*.

### 3.5. TF Activity Analyzer Based on Differential Accessibility Analysis

We developed the TF activity analyzer to study whether a TF is active at per-cell or per-nucleotide resolution. Specifically, the TF activity analyzer comprises two functions: single-cell TF activity inference and single-nucleotide TF activity.

**Single-cell TF activity inference based on motif insertion.** To compute a TF activity score for each TF for each cell, we performed motif insertion on PROTRAIT. Specifically, we first performed dinucleotide shuffling of randomly sampled scATAC-seq peaks to generate genomic background sequences. For each TF, we downloaded the motif sequences from the JASPAR database and inserted them into the center of each background sequence. Then, we predicted normalized accessibility across all cells for both motif-inserted sequences and background sequences by PROTRAIT. Finally, we took the difference in predicted accessibility between motif-inserted sequences and background sequences as the motif influence score for each sequence. For each cell, the averaged influence score across all sequences can be regarded as a cell-level prediction of TF activity.

**Single-nucleotide TF activity inference based on ISM.** To further compute a TF activity score at per-cell per-nucleotide resolution, we performed ISM for all single nucleotides of a DNA sequence. We first calculated the change in accessibility in every cell after mutating each position to its three alternative nucleotides. Then, we normalized the changed accessibility for the four nucleotides at each position such that they sum to zero. For a motif sequence, the normalized score at the reference nucleotide can be regarded as a nucleotide-level prediction of TF activity.

## 4. Conclusions

In this paper, we developed a deep learning framework based on the ProdDep Transformer Encoder, called PROTRAIT, for scATAC-seq analysis. Compared to previous approaches for single-cell data analysis, PROTRAIT has three distinct characteristics: (i) it utilizes the ProdDep Transformer Encoder to capture occupancy, position, and long-range dependency of TF-DNA binding motifs from scATAC-seq peaks; (ii) it incorporates chromatin accessibility prediction, cell type annotation, scATAC-seq data denoising, and TF activity inference into a unified framework; and (iii) it is easily scalable to large-scale single-cell data analysis accelerated using GPU parallelism.

We applied PROTRAIT to scATAC-seq datasets from the Buenrostro study and comprehensively compare its performance with state-of-the-art analysis pipelines. Our experimental results demonstrate that PROTRAIT outperforms state-of-the-art channels in chromatin accessibility prediction, cell type annotation, and scATAC-seq data denoising. Furthermore, we designed in silico experiments and validated that PROTRAIT can query for TF motif activity in single cells and single nucleotides. Finally, we confirmed the scalability of PROTTAIT to million-cell datasets.

Several directions are foreseen to improve further and expand our approach. First, we only used DNA sequences from the reference genome; however, many sequences may have structural variations, such as insertions, deletions, and inversions, leading our approach astray. Thus, employing single-cell chromatin accessibility and structural variation data simultaneously to train our system may lead to better performance. Second, integrating multiple molecular features from various modalities, such as chromatin accessibility, gene expression, and protein abundance, is another promising research direction. 

## Data Availability

All the data analyzed during the current study are available in http://storage.googleapis.com/scbasset_tutorial_data/buen_ad_sc.h5ad (accessed on 23 December 2022) and http://renlab.sdsc.edu/kai/Key_Processed_Data/Cell_by_cCRE (accessed on 24 November 2021). The codes are available at https://github.com/ZhangLab312/PROTRAIT (accessed on 4 January 2023).
